# The role of oral metformin in preventing and treating age-related macular degeneration: A meta-analysis

**DOI:** 10.1097/MD.0000000000038728

**Published:** 2024-07-12

**Authors:** Rowan H. Elhalag, Mai Saad Mohamed, Marwan Abowafia, Marina Ramzy Mourid, Nada Mahmoud, Youmna Abourady, Paula Ghali, Momen Hassan Moussa, Jaffer Shah, Karam R. Motawea

**Affiliations:** a Faculty of Medicine, Alexandria University, Alexandria, Egypt; b Weill Cornell Medicine, New York, NY, USA.

**Keywords:** age-related macular degeneration, AMD, metformin

## Abstract

**Background:**

We aimed to perform a meta-analysis to evaluate the effect of metformin on age-related macular degeneration.

**Methods:**

We searched the following databases: PubMed, Scopus, and Web of Science. We included any randomized control trials, prospective and retrospective cohorts, cross-sectional studies, and case–control studies that investigated the effect of metformin on age-related macular degeneration in our meta-analysis with no age or language restrictions. Review manager software, version 5.4 was used to perform the meta-analysis.

**Results:**

Ten studies were included in the meta-analysis with 1,447,470 patients included in the analysis. The pooled analysis showed no statistically significant difference between the metformin group and the non-metformin group regarding age-related macular degeneration (odds ratio [OR] = 0.37, confidence interval [CI] = (0.14–1.02), *P* = .05). Subgroup analysis showed no statistically significant difference between metformin group and non-metformin group regarding age-related macular degeneration in present or past metformin usage (OR = 0.19, CI = (0.03–1.1), *P* = .06), (OR = 0.61, CI = (0.25–1.45), *P* = .26), respectively, The pooled analysis showed no statistically significant difference between age-related macular degeneration group and control group regarding metformin usage (OR = 0.86, CI = (0.74–1.00), *P* = .05). The subgroup analysis showed no statistically significant difference between the age-related macular degeneration group and control group in <2 years of metformin usage and 2 years or more (OR = 0.89, CI = (0.52–1.52), *P* = .67), (OR = 0.95, CI = (0.82–1.10), *P* = .47), respectively.

**Conclusion:**

Our study revealed no role of metformin in decreasing age-related macular degeneration risk in past or present usage. More RCTs are needed to support our findings in evaluating the actual role of metformin in age-related macular degeneration.

## 1. Introduction

Age-related macular degeneration (AMD) is a degenerative eye disease known to be the primary cause of irreversible vision impairment in people over the age of 50 throughout the world. The presence of drusenoid deposits characterizes early AMD, whereas late AMD entails atrophy of the retinal pigment epithelium or the development of choroidal neovascularization. AMD is linked to several risk factors, including cigarette smoking, obesity, hypertension, and high cholesterol levels.^[1–3]^ AMD is predicted to affect approximately 196 million individuals worldwide, with a projected increase to 288 million by 2040.^[4]^ It is estimated that over 8 million people in the UK have some type of AMD^[5]^; approximately 600,000 people struggle with sight loss caused by AMD, with approximately 70,000 new cases each year.^[6]^ AMD costs the UK economy £2.6 billion each year, with more than half of that (53%) falling outside of health and social care,^[7]^ and prescription expenses exceeding £500 million.^[8]^ At the current rate, there will be 1.3 million persons with AMD by 2050. With an aging population, it is critical that new treatments are developed, ideally to stop AMD in its early, non-sight-threatening phases or to offer protection against the onset of AMD. As a result, researchers are currently focused on evaluating preventive measures for the development of AMD.^[9]^

Metformin, a biguanide that is indicated as a first-line diabetes treatment for type 2 diabetes mellitus, has anti-inflammatory and antioxidant properties^[10,11]^ and reduces blood glucose levels. As a result, whether metformin use influences the risk of age-related eye illnesses AMD and diabetic retinopathy, which are of public health importance, warrants additional investigation. Metformin has long been claimed in laboratory experiments to have “anti-aging” properties.^[11]^ Several biological reasons exist to investigate the influence of metformin on the risk of incident AMD: first, the metabolic environment of the eye is severe, and as a result, resilience may be limited^[12]^; second, linkages between AMD and metabolic dysregulation have been found in a variety of study scenarios, including animal models of AMD (including light-induced photoreceptor cell loss),^[13]^ AMD genetic studies (dry and wet types),^[14,15]^ as well as in systemic profiles of AMD patients showing oxidative stress.^[16]^ Third, metformin has been shown in animal models to promote glucose utilization in the retina while also protecting photoreceptors and the retinal pigment epithelium against oxidative stress.^[17]^ Fourth, metformin appears to function via AMPK, mutations of which have been linked to photoreceptor destruction and “accelerated aging” phenotypes.^[18]^

Recently, research has focused on determining the true impact of metformin use on AMD. Some research found that metformin protects against AMD, whereas others found the opposite. Furthermore, other studies found no effect at all. A recently published meta-analysis reported that metformin might play a crucial role in decreasing the risk of AMD.^[19]^ As a result, we conducted this meta-analysis to clarify this discrepancy and determine the true impact of metformin on AMD.

## 2. Methods

The Cochrane Handbook of Systematic Reviews, The PRISMA 2020 Update, The MOOSE Guidelines, and the standards for Preferred Reporting Items of Systematic Reviews and Meta-Analyses were all followed when conducting this review. (A PRISMA 2020 Checklist completed form was submitted).^[20]^

### 2.1. Study design

A meta-analysis study aimed to determine the role of metformin as a prophylactic therapy against AMD.

### 2.2. Search strategy

We searched 3 databases: PubMed, Scopus, and WebOf Science (WOS) on February 2, 2023, using MeSH terms to form the following search strategy: “(Metformin OR Dimethylbiguanidine OR Dimethylguanylguanidine OR Glucophage) AND (Age-related macular degeneration OR Maculopathies OR maculopathy OR Macular Dystrophy)” in PubMed, while “(“Metformin” OR “Glucophage” OR “Dimethylbiguanidine” OR “Dimethylguanylguanidine”) AND (“Age-related macular degeneration” OR “Maculopathies” OR “maculopathy” OR “Macular Dystrophy”)” was used in the rest of the databases. As a secondary check, 2 authors manually searched the references of the included studies.

### 2.3. Study selection

Our inclusion criteria were any original studies; randomized control trials prospective, or retrospective cohort, cross-sectional and case–control studies that investigated the efficacy of metformin usage in elderly diabetic patients suffering from AMD published in the English language was included in our meta-analysis with no age restrictions.

We adhered to a specific set of criteria outlined by PICO: (1) patients with AMD; (2) metformin usage (whether present use during the study or using it in the past and not during the study); (3) placebo or no drug at all; (4) effect on already existing AMD or the risk of developing of a non-existing AMD. Our selection process excluded missing PICO, case reports, case series, editorials, review papers, animal-based studies, and secondary reviews.

Two authors conducted the screening and data collection. Screening passed by 2 processes: (1) title and abstract screening and (2) full-text screening. Two authors from each performed title and abstract screening of each study using the Covidence platform, the study was included if it seemed eligible for inclusion according to our inclusion criteria, then the same 2 authors performed full-text screening of each study included from the title and abstract screening phase. The first author resolved the disputes and compared the results from the 2 groups. The study was finally included in our analysis at this phase if it met our inclusion criteria.

### 2.4. Data extraction

Two Excel sheets were created from the data we extracted from the included studies; in the first one, one author extracted baseline characteristics: age, sex, comorbidities, medical conditions, medications, the number of patients in each group, Charlson Comorbidity Index, and race/ethnicity and the other contained: the study design, duration of the study, study arms, endpoints, and conclusion.

### 2.5. Quality assessment

The New Castle Ottawa Scale tool^[21]^ was used to assess the quality of the included studies. Each study was given a score and ranked as; good, fair, or poor quality.

### 2.6. Data analysis

Review Manager Software version 5.4 was used to perform the meta-analysis; the dichotomous outcomes were measured as odds ratio (OR) and confidence interval (CI) and the generic inverse variance was used to measure the overall adjusted odds ratio of the outcomes with a 95% confidence interval. In case of heterogeneity (Chi-square *P* value < .05), a random effect model was adopted, otherwise, a fixed-effect model was employed, in general; the results were considered significant if the *P*-value was <.05.

## 3. Results

**Figure 1.**
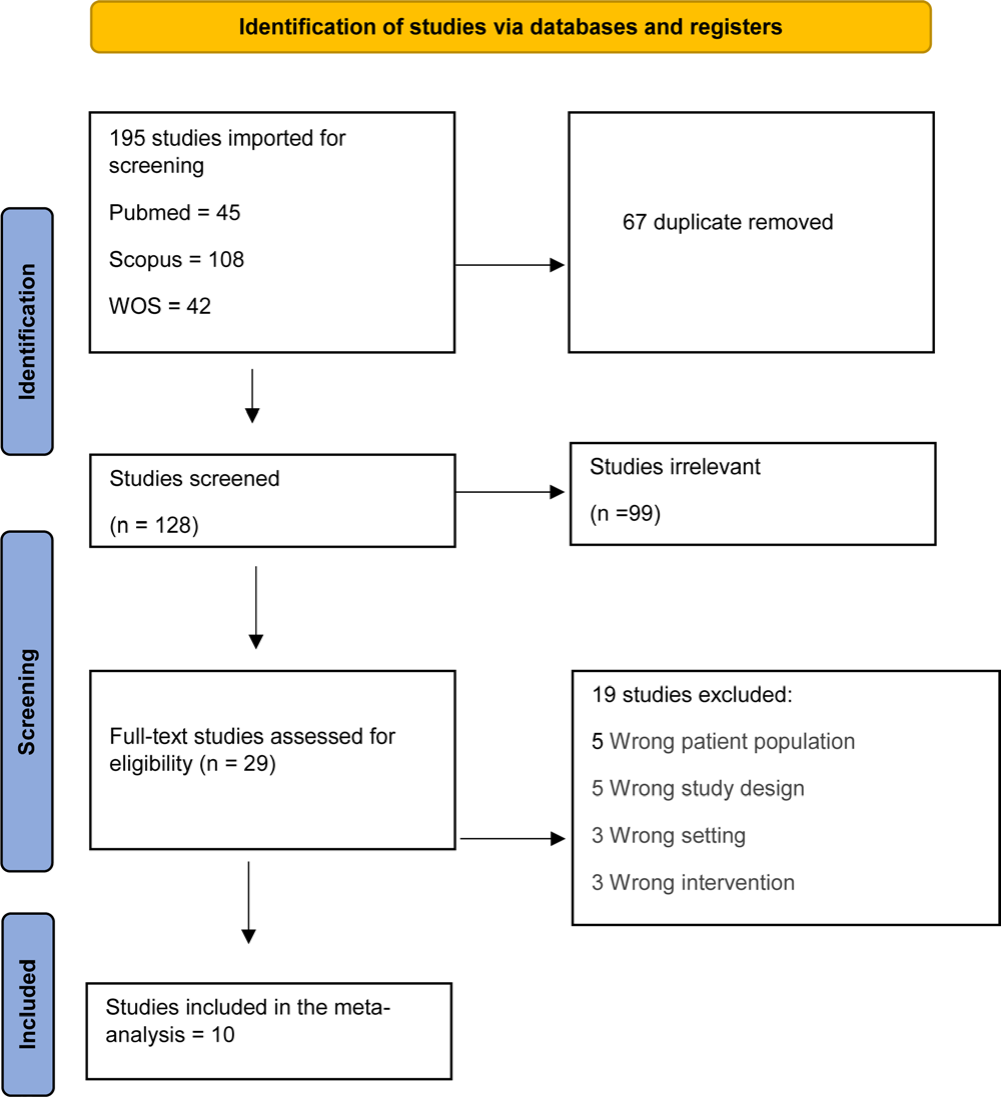
PRISMA flow diagram.

After a complete literature search, 195 studies resulted and then 128 became eligible for title and abstract screening after removal of duplicates. Of the 128, 99 were irrelevant and 29 studies were eligible for full-text screening. Finally, 10 studies were included in the meta-analysis after full-text screening, as shown in the PRISMA (Fig. 1). A summary of the included studies is shown in Table 1.

**Table 1:** Summary of the included studies.

ID	Study design	Study arms	Study conclusion	Quality assessment
Blitzer et al 2021^[22]^	A case-control sudy	Case Group: adults aged 55 years and older who were newly diagnosed with AMD Control Group: Healthy adults selected from the general population represented in MarketScan data	This study showed that metformin use was associated with reduced odds of developing AMD. This association was dose dependent, with the greatest benefit at low to moderate doses. Metformin does not appear to be protective in patients with diabetes and coexisting diabetic retinopathy.	Good
Brown et al 2019^[23]^	A retrospective case- control study	Case Group: adults diagnosed with AMD Control Group: Healthy adults	This study showed that patients who had taken metformin had decreased odds of developing AMD, suggesting that metformin may have a therapeutic role in AMD development or progression in those who are at risk.	Good
Chen et al 2019^[24]^	A retrospective cohort study	Case Group: Type 2 diabetes patients receiving metformin Control Group: Type 2 diabetes patients not receiving metformin	This study showed that among patients with type 2 diabetes, those who use metformin are at a significantly lower risk of developing AMD relative to individuals who do not use metformin. Also, the trend of a significantly lower AMD risk was found with a higher dose of metformin.	Good
Eton et al 2022^[25]^	A retrospective cohort study	Case Group: Type 2 diabetes patients currently receiving metformin Control Group: Type 2 diabetes patients not currently receiving metformin (past use)	This study showed small, conflicting associations between metformin exposure and development of dry AMD, suggesting that metformin did not substantially affect the development of dry AMD.	Good
Gokhale et al 2022	A population-based retrospective open cohort study with a time- dependant exposure design.	Case Group: Type 2 diabetes patients receiving metformin Control Group: Type 2 diabetes patients not receiving metformin	This study showed no evidence that metformin was associated with risk of age-related macular degeneration in primary care patients requiring treatment for type 2 diabetes.	Good
Jiang et al 2022^[26]^	A retrospective cohort study	Case Group: Type 2 diabetes patients receiving metformin Control Group: Type 2 diabetes patients not receiving metformin	This study showed that among patients with T2DM for ≥ 10 years, metformin users were less likely to develop any AMD and early AMD than non-users; however, the late AMD was not significantly associated with the use of metformin. Also, AMD was less prevalent in patients with diabetic retinopathy. The prolonged metformin treatment with a high cumulative dose enhanced the protective effect against AMD. Metformin significantly reduces the AMD risk when the cumulative duration is > 5 years.	Good
Lee et al 2019^[27]^	A nested case-control study	Case Group: adults diagnosed with AMD Control Group: Healthy adults	This study showed that statins, metformin, ACE inhibitors, and ARBs did not inhibit AMD in elderly patients. The absence of a duration-response supports the lack of a causal relationship.	Good
Shaw et al 2022	A case-control study	Case Group: adults aged 55 years and older who were newly diagnosed with AMD Control Group: Healthy adults selected from the general population represented in MarketScan data	This study showed that metformin, insulin, and sulfonylureas are protective against the development of AMD in diabetic patients. Other diabetic medications may place diabetics at a higher risk of developing AMD, but this risk is alleviated when taken in combination with metformin.	Poor
Stewart et al 2020^[28]^	A cross-sectional retrospective study	Case Group: adults diagnosed with AMD Control Group: Healthy adults	This study demonstrated that oral metformin use was associated with a reduced odds of AMD, with consistent results for the overall AMD outcome and the non-neovascular AMD outcome.	Good
Vergroesen et al 2022^[29]^	A cohort study	Case Group: Type 2 diabetes patients receiving metformin Control Group: Type 2 diabetes patients not receiving metformin	This study showed that although diabetes was clearly associated with cataract, diabetes medication was not.	Poor

A total of 10 studies were included, 5 of which were retrospective cohorts, 4 case-controls and one was cross-sectional study. The quality of observational studies was assessed using New Castle Ottawa Scale. All of the included studies were rated as good quality in both the selection (scores = 3), and outcome (scores = 2) domains. Only the comparability domain showed some variation (score ≤ 1). The overall quality was good in 8 studies^[22–28,30]^ and poor in 2 studies^[29,31]^ as shown in the supplementary material, http://links.lww.com/MD/N200.

The total number of patients included in the study is 1,447,470 patients, 451,551 patients in the case group, and 995,919 patients in the control group. Other baseline data are shown in Table 2.

**Table 2:** Baseline characteristics of the included studies.

ID	Number of patients in each group			Age (Years) M,SD				sex (n)						Comorbidites & Medical conditions		Medications		HbA1c, %		Charlson Comorbidity Index (CCI) Mean, SD				Race/ethinity N (%)			
	Cases	Controls		Cases		Controls		Cases			Controls																
							Female		Male	Female		Male	Cases	Controls	Cases		Controls	Cases	Controls	Cases		Controls		Cases		Controls	
Blitzer 2021^[22]^	312 404	312 376	74.9 (10.3)		74.9 (10.3)		181 817		130 587	172 459		139 917	Diabetes 81 262 Smoking 17 841 Hyperlipidemia 155 080 Hypertension 203 463 Anemia 20 197 Non proliferative DR 11 400 Proliferative DR 2241	Diabetes 79 497 Smoking 12 920 Hyperlipidemia 149 627 Hypertension 203 475 Anemia 20 122 Non proliferative DR 5281 Proliferative DR 1229	Metformin 40,081 Insulin 18,257 Sulfonureas 25,775 Glitazones 9619 Meglitinides 1938 Other diabetes medications 12,089 Statins 162,856		Metformin 40,730. Insulin 17,383 Sulfonureas 26,702 Glitazones 9948 Meglitinides 1861 Other diabetes medications 11,262 Statins 165,393	NA	NA	1.1 (1.5)		1.1 (1.5)		NA	NA	NA	NA
Brown 2019^[23]^	1947	5841	median (SD)= 77 (10.10)		median (SD) =75 (8.55)		1197		750	3039		2802	Diabetes mellitus 837 Detached retina 37 Macular cyst, hole, or pseudohole 79 Puckered macula 265 Drusen 110 Retinal hemorrhage 50 Retinal exudates or deposits 9 Retinal edema 162 Retinal ischemia 19	Diabetes mellitus 4110 Detached retina 3 Macular cyst, hole, or pseudohole 42 Puckered macula 192 Drusen 32 Retinal hemorrhage 8 Retinal exudates or deposits 1 Retinal edema 30 Retinal ischemia 8	Metformin 85 Alpha inhibitors 0 DPP4 inhibitors 19 Meglitinides 0 SGLT2 inhibitors 2 Thiazolidinediones 1 Serotonin modulators and simulators 1 Serotonin-norepinephrine reuptake inhibitors 1 SSRI 28 Tetracyclic antidepressants 32 Statins 270		Metformin 610 Alpha inhibitors 3 DPP4 inhibitors 169 Meglitinides 5 SGLT2 inhibitors 21 Thiazolidinediones 4 Serotonin modulators and simulators 3 Serotonin-norepinephrine reuptake inhibitors 8 SSRI 67 Tetracyclic antidepressants 64 Statins 973	NA	NA	4.26 (3.71)		4.29 (3.59)		Caucasian 1583 Black/African American 245 Other racial minority 119 Unknown 0	Unknown 0	Caucasian 4431 Black/African American 983 Other racial minority 384	Unknown 43
Chen 2019^[24]^	45,524	22,681	55.2 ± 12.6		57.8 ± 12.7		20,753		24,771	11,560		11,121	N (%) Hypertension 32,004 (70.3) Hyperlipidemia 31,575 (69.4) Coronary artery disease 14,481 (31.8) Obesity 2578 (5.7) Diabetic retinopathy 5294 (11.6) Chronic kidney disease 5444 (12.0)	N (%) Hypertension 14,517 (64.0) Hyperlipidemia 13,486 (59.5) Coronary artery disease 6357 (28.0) Obesity 927 (4.1) Diabetic retinopathy 433 (1.9) Chronic kidney disease 5399 (23.8)	N (%) Insulin 1457 (3.2) Sulfonylurea 25,448 (55.9) DDP-4 inhibitor 6738 (14.8) thiazolidinedione 6510 (14.3) Meglitinide 2189 (4.8) α-glucosidase inhibitor 2232 (4.9) Antihypertensives 27,953 (61.4) Lipid-lowering agents 26,994 (59.3)		N (%) Insulin 1429 (6.3) Sulfonylurea 14,176 (62.5) DDP-4 inhibitor 3674 (16.2) thiazolidinedione 2746 (12.1) Meglitinide 2135 (9.4) α-glucosidase inhibitor 1858 (8.2) Antihypertensives 12,631 (55.7) Lipid-lowering agents 12,840 (56.6)	NA	NA	NA	NA	NA	NA	NA	NA	NA	NA
Gokhale 2022	173,689		Males: 61.9 (11.2), Females: 64.0 (11.9)				male = 99,093/female = 74,596						Male N (%): Peripheral Neuropathy 5512 (5.6%) Sight Threatening Retinopathy 1930 (1.9%) Foot ulcer or amputation 2207 (2.2%) Hypothyroidism 3349 (3.4%) Cardiovascular disease 26,243 (26.5%) female N (%): Peripheral Neuropathy 3674 (4.9%) Sight Threatening Retinopathy 1579 (2.1%) Foot ulcer or amputation 1852 (2.5%) Hypothyroidism 10,656 (14.3%) Cardiovascular disease 14,616 (19.6%)		Male N (%) Other Antidiabetic Drugs only 10,809 Metformin only or in combination with other antidiabetic medications 87,830. No drug 454 Statin prescription 60,978. Female N(%) Other Antidiabetic Drugs only 8016 Metformin only or in combination with other antidiabetic medications 66,186 No drug 394 Statin prescription 43,025			Male Hba1c = N(%) <6.5 (<48) = 6314 (6.4%) 6.5–7 (48–53) = 7995 (8.1%) 7–7.5 (53–58.5) = 11,348 (11.5%) 7.5–8 (58.5–64) = 12,934 (13.1%) 8–8.5 (64–69.5) = 7410 (7.5%) 8.5 + (69.5+) = 35,166 (35.5%) Missing = 17,926 (18.1%) Female Hba1c = N(%) <6.5 (<48) = 5208 (7.0%) 6.5–7 (48–53) = 7372 (9.9%) 7–7.5 (53-58.5) = 9630 (12.9%) 7.5–8 (58.5–64) = 10,372 (13.9%) 8–8.5 (64–69.5) = 5680 (7.6%) 8.5 + (69.5+) = 22,770 (30.5%) Missing = 13,564 (18.2%)		Male CCI = N(%) 0 = 56,995 (57.5%) 1 = 23,663 (23.9%) 2+ =18,435 (18.6%) Female CCI = N(%) 0 = 40,876 (54.8%) 1 = 19,123 (25.6%) 2+ = 14,597 (19.6%)				Male N (%) White 43,369 (43.8%) Black 1337 (1.3%) South Asian 2893 (2.9%) Mixed Race 656 (0.7%) Other ethnicity 215 (0.2%) Missing 50,623 (51.1%) Female N(%) White 32,226 (43.2%) Black 1216 (1.6%) South Asian 2519 (3.4%) Mixed Race 538 (0.7%) Other ethnicity 185 (0.2%) Missing 37,912 (50.8%)			
Eton 2022^[25]^	1007,226		67.5 (8.9)				Female = 536,120 (53.3%) Male = 471,106						NPDR 80,003 (7.9%) Hypertension 869,713 (86.4%) Hypercholesterolemia 867,060 (86.1%) Kidney disease CKD 201,993 (20.1%) ESRD 15,434 (1.5%) Anemia 79,697 (7.9%)		Statin 350,431 (34.8%) Insulin use 54,518 (5.4%) Metformin Current use 166,115 (16.5%) Cumulative dose (mg) 162,859.3 (383,963.8)			6.8 (1.4)		NA				White 668,580 (66.4%) Asian 33,151 (3.3%) Black 127,669 (12.7%) Hispanic 89,388 (8.9%) Other/unknown 87,694 (8.7%)			
Jiang 2022^[26]^	209	115	66 (61.0, 75.0)		68 (63.0, 77.0)		91		118	58		57	Hypertension 134 Hyperlipidemia 103 Diabetic retinopathy 61	Hypertension 76 Hyperlipidemia 38 Diabetic retinopathy 30	Insulin users 102 (48.8%)		Insulin users 59 (51.3%)	7.8 (6.8, 9.2)	7.3 (6.1, 8.8)	NA	NA	NA	NA	NA	NA	NA	NA
Lee 2019^[27]^	2330	23,278	66.5 ± 5.0		66.4 ± 5.0		1471		859	14,710		8568	Cerebrovascular diseases 941 (40.4) Complicated diabetes mellitus 260 (11.2) Uncomplicated diabetes mellitus 501 (21.5) Hyperlipidemia 43 (1.8) Hypertension 0 (0.0) Liver diseases 32 (1.4) Myocardial infarction 32 (1.4) Peripheral vascular diseases 489 (21.0)	Cerebrovascular diseases 8253 (35.5) Complicated diabetes mellitus 2210 (9.5) Uncomplicated diabetes mellitus 4324 (18.6) Hyperlipidemia 375 (1.6) Hypertension 3 (0.0) Liver diseases 325 (1.4) Myocardial infarction 257 (1.1) Peripheral vascular diseases 4245 (18.2)	Alpha-blockers 987 (42.4) Alpha-glucosidase 536 (23.0) Aspirin 25 (1.1) Beta-blockers 119 (5.1) Calcium channel blockers 70 (3.0) Diuretics 1034 (44.4) Meglitinide 1358 (58.3) Sulfonylurea 743 (31.9) Thiazolidinedione 138 (5.9)		Alpha-blockers9278 (39.9) Alpha-glucosidase 4879 (21.0) Aspirin 25 (1.1) Beta-blockers 119 (5.1) Calcium channel blockers 465 (2.0) Diuretics 9739 (41.8) Meglitinide 12,676 (54.5) Sulfonylurea 6602 (28.4) Thiazolidinedione 1357 (5.8)	NA	NA	0.9 ± 0.6		0.8 ± 0.6		NA	NA	NA	NA
Shaw 2022	81,262	79,497	NA		NA		NA		NA	NA		NA	NA	NA	NA		NA	NA	NA	NA	NA	NA	NA	NA	NA	NA	NA
Stewart 2020^[28]^	148	2972	79.5 ± 10.3		72.1 ± 9.1		82		66	1561		1405	NA	NA	Metformin 68 Insulin 46 Sulfonylureas 50 DPP-4 inhibitors 16 Thiazolidinediones 8 Meglitinides 6 Alpha-glucosidase inhibitor 5 SGLT-2 inhibitors 2 Bile acid sequestrants 1		Metformin 1739 Insulins 1007 Sulfonylureas 875 DPP-4 inhibitors 295 Thiazolidinediones 283 Meglitinides 79 Alpha-glucosidase inhibitor 35 SGLT-2 inhibitors 23 Bile acid sequestrants 9	NA	NA	NA	NA	NA		White 59 Black 8 Asian 59 Hispanic 14 Other 22		White 910 Black 324 Asian 1081 Hispanic 359 Other 657	
Vergroesen 2022^[29]^	788	751	NA		NA		380		408	400		351	NA	NA	Metformin 617 sulfonylurea 125 insulin 39 combination treatment 7 Antihypertensives 353 Statins 150		Antihypertensives 231 Statins 59	NA	NA	NA	NA	NA	NA	NA	NA	NA	NA

## 4. Outcomes

### 4.1. AMD development

**Figure 2.**
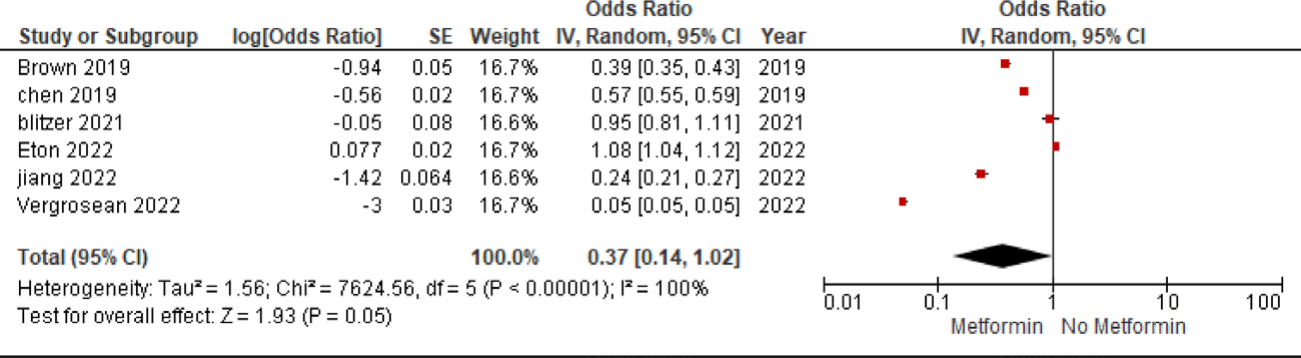
AMD risk after metformin use. AMD = age-related macular degeneration.

The pooled analysis showed no statistically significant difference between the metformin group and the non-metformin group (OR = 0.37, CI = (0.14–1.02), *P* = .05). We observed significant heterogeneity among studies (*P* < .00001, I² = 100%) that was not solved by leaving one out test, as shown in Figure 2.

### 4.2. Current status of metformin usage in AMD patients and its effect on AMD subgroup analysis

#### 4.2.1. Present

**Figure 3.**
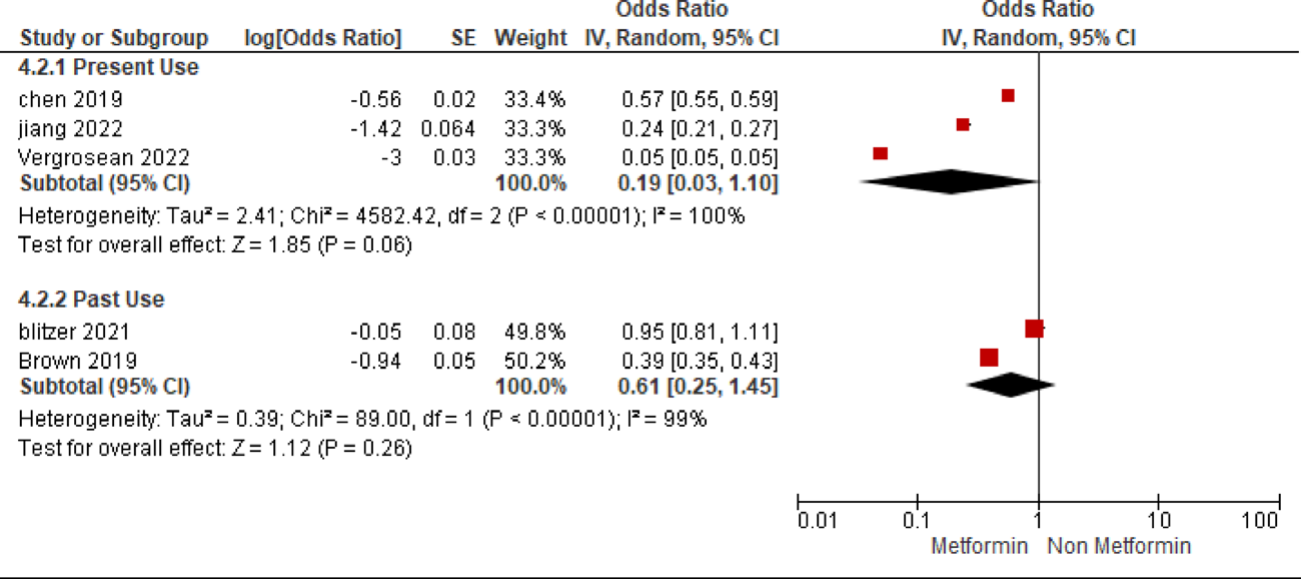
Current status of metformin usage in AMD patients and its effect on AMD subgroup analysis. AMD = age-related macular degeneration.

The pooled analysis showed no statistically significant difference between individuals who were administered metformin and those who were not (OR = 0.19, CI = (0.03–1.1), *P* = .06). We observed significant heterogeneity among studies (*P* < .00001, I² = 100%) which persisted even after conducting the leave-one-out test, as shown in Figure 3.

#### 4.2.2. Past

The pooled analysis showed no statistically significant difference between patients receiving metformin and patients not receiving metformin (OR = 0.61, CI = (0.25–1.45), *P* = .26). We observed significant heterogeneity among studies (*P* < .00001, I² = 99%) that was not solved by leave one out test, as shown in Figure 3.

#### 4.2.3. Metformin usage

**Figure 4.**
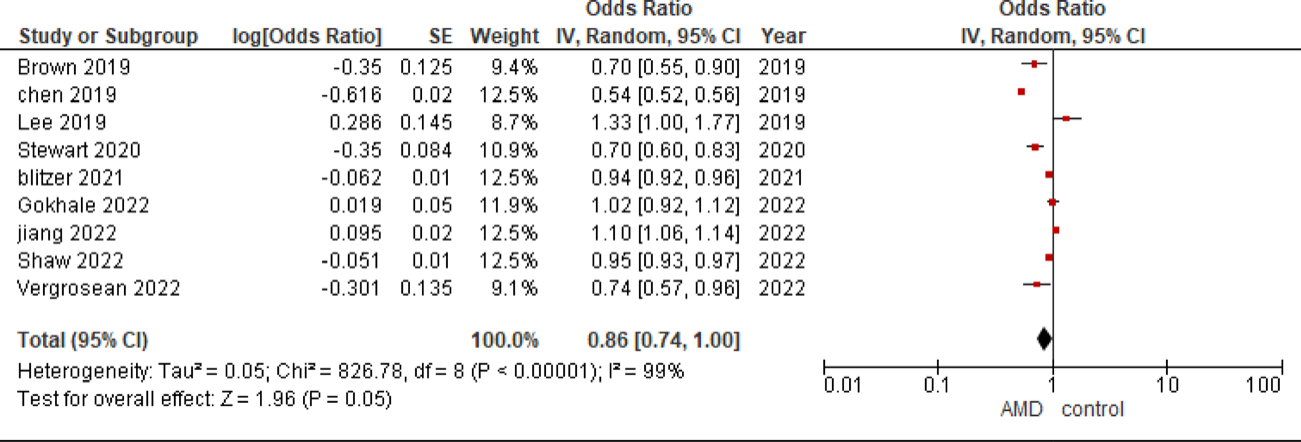
Metformin usage in patients with already existing AMD. AMD = age-related macular degeneration.

The pooled analysis showed no statistically significant difference between patients receiving metformin and patients not receiving metformin (OR = 0.86, CI = (0.74–1.00), *P* = .05). We observed significant heterogeneity among studies (*P* < .00001, I² = 99%) that was not solved by leave one out test, as shown in Figure 4.

### 4.3. Current status of metformin use in healthy patients and the risk of AMD subgroup analysis

#### 4.3.1. Present

**Figure 5.**
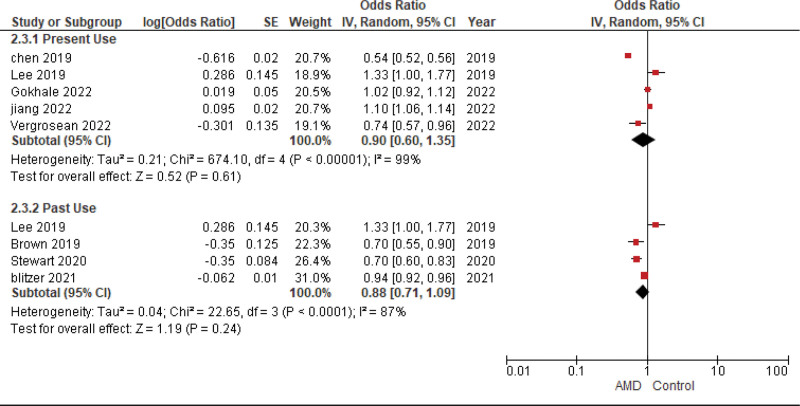
Current status of metformin use in healthy patients and the risk of AMD subgroup analysis. AMD = age-related macular degeneration.

The pooled analysis showed no statistically significant difference between patients receiving Metformin and patients not receiving metformin (OR = 0.90, CI = (0.60–1.35), *P* = .61). We observed significant heterogeneity among studies (*P* < .00001, I² = 99%) that was not solved by leave one out test, as shown in Figure 5.

#### 4.3.2. Past

The pooled analysis showed no statistically significant difference between patients receiving Metformin and patients not receiving metformin (OR = 0.88, CI = (0.71–1.09), *P* = .24). We observed significant heterogeneity among studies (*P* < .0001, I² = 87%) that was not solved by leave one out test, as shown in Figure 5.

#### 4.3.3. Duration of usage subgroup analysis

##### 4.3.3.1. Less than 2 years

**Figure 6.**
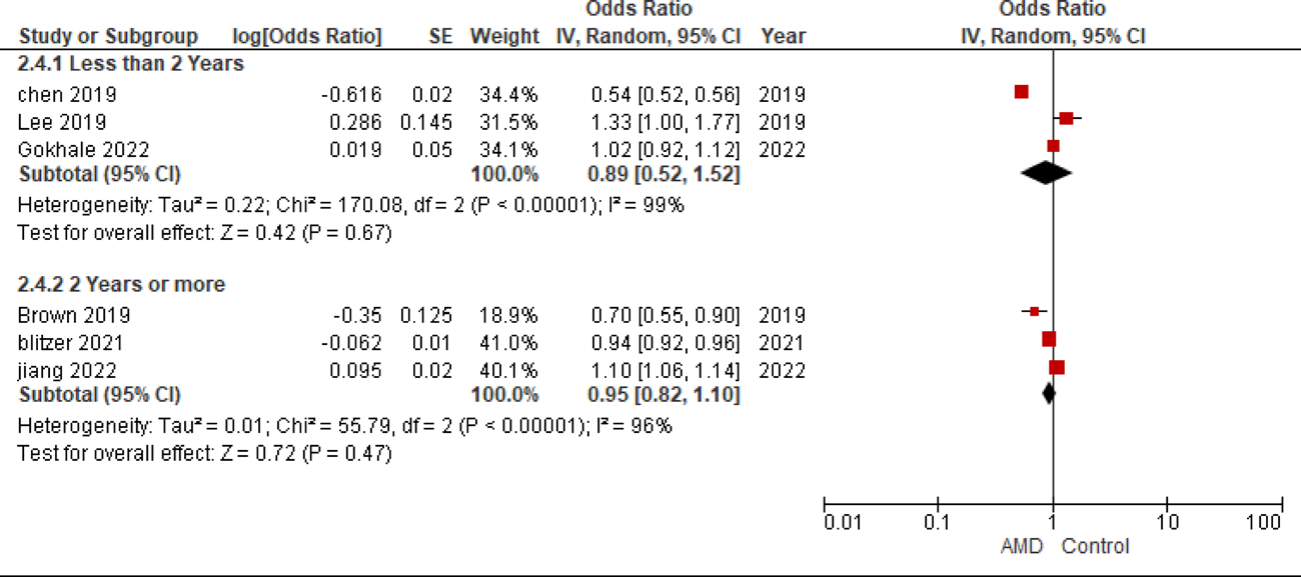
Duration of metformin usage subgroup analysis.

The pooled analysis showed no statistically significant difference between patients receiving Metformin and patients not receiving metformin (OR = 0.89, CI = (0.52–1.52), *P* = .67). We observed significant heterogeneity among studies (*P* < .00001, I² = 99%), so we performed leave one out test by removing the study^[24]^, and the heterogeneity was solved (*P* = .08, I² = 67%) and the result showed no statistically significant difference between metformin group and non-metformin group (OR = 1.13, CI = (0.87–1.45), *P* = .36), as shown in Figure 6.

##### 4.3.3.2. Two years or more

The pooled analysis showed no statistically significant difference between patients receiving metformin and patients not receiving metformin (OR = 0.95, CI = (0.82–1.10), *P* = .47). We observed significant heterogeneity among studies (*P* < .00001, I² = 96%) that was not solved by leave one out test, as shown in Figure 6.

## 5. Discussion

On analyzing 10 papers, our analysis revealed no significant difference in the occurrence of AMD between patients who were prescribed metformin and those who were not. Additionally, there was no notable distinction between the AMD group and the healthy control group when both received metformin. Subgroup analyses were conducted based on the current usage of metformin, including past and present use, revealing no significant differences between patients taking metformin and those not taking it in both healthy and AMD patient populations. Furthermore, another subgroup analysis was performed concerning the duration of metformin usage, the analysis indicated that there was no notable distinction between individuals using metformin and those not using it, irrespective of whether the duration of usage was <2 years or equal to or >2 years.

Previous studies conducted in the United States and East Asia yielded inconsistent results. However, our study aligns with the findings of a nested case–control study conducted in South Korea.^[27]^ The researchers concluded that the consumption of metformin did not lead to a reduced risk of AMD in the older age group. Additionally, no significant associations were found between the risk of AMD and the total duration or timing of metformin use. The study exhibited several strengths. Firstly, the utilization of NHIS-NEC data from a well-established and validated national longitudinal database, covering follow-up data for 558,147 older individuals from 2002 to 2015, added robustness to their findings. Secondly, the inclusion of data from the entire population of South Korea enhanced the external validity and generalizability of the study, making the results applicable to real-world scenarios. Thirdly, the study accounted for various potential confounding factors, such as socioeconomic status, healthcare resource utilization, combination drug use, and comorbidities. However, certain limitations were acknowledged. AMD cases were identified based on diagnoses recorded in the database, which might not perfectly align with the actual diagnoses received by the patients. Additionally, the study could not include variables related to lifestyle, family history, and cardiovascular factors (e.g., blood pressure, HbA1c, and lipid profile) due to the absence of this information in the database. These factors are recognized as potential risk factors for AMD, and their exclusion could impact the comprehensiveness of the study’s findings. Moreover, the findings by Vergroesen et al indicated that, although diabetes was associated with cataracts, the use of diabetes medication did not show the same association.^[29]^ Specifically, the use of Metformin was correlated with a reduced risk of open-angle glaucoma but did not demonstrate a similar association with AMD. Interestingly, other diabetes medications, such as insulin and sulfonylurea derivatives, were associated with a decreased risk of AMD. On the contrary, a case–control study conducted by Blitzer et al^[22]^ reported that the use of metformin for over 2 years in individuals aged 55 and older was associated with a 5% to 10% lower odds ratio of developing AMD in a large national sample comprising 312,404 AMD patients. However, this study has several limitations. Firstly, it was unable to establish the actual likelihood of developing AMD; instead, it presented odds ratios associating metformin use with AMD. Secondly, the research relied on diagnosis codes extracted from billing records, raising the possibility that diagnoses might be underreported or inaccurately recorded. For instance, mild AMD might be documented as drusen, and since their study excluded cases without an additional AMD code, peripheral retinal drusen cases were omitted. Thirdly, the MarketScan database utilized in the study only encompassed information on patients with private insurance, which may not accurately represent the elderly population. Moreover, a retrospective propensity score matched cohort study involving individuals with type 2 diabetes, conducted using the Taiwan National Database, revealed that individuals using metformin had a significantly lower risk of developing AMD compared to nonusers.^[24]^ However, it is important to note the impending limitations of the study. The index date was defined as the date of type 2 diabetes diagnosis among metformin users. Consequently, the period between the diagnosis of type 2 diabetes and the initiation of metformin may have been inaccurately classified as exposed time, introducing a potential source of bias known as eternal time bias. This bias arises when the classification of exposure time is not accurately represented, leading to an overestimation of the preventive effect. The study suggests that metformin’s preventive effect on AMD is dose- and duration-dependent. The observed association may be attributed to eternal time bias, where patients classified as high total dose or long-duration metformin users must have been free of AMD for a longer period to be prescribed high doses of metformin or exposed for extended durations. Surprisingly, Eton et al^[25]^ discovered that current metformin use during the study period was associated with a slight yet significant increase in the risk of developing dry AMD. However, when past metformin use (rather than present) was considered, a contrasting protective correlation was observed. Additionally, advancing age was found to be linked to a higher likelihood of developing dry AMD. While evaluating the findings of this study, it is essential to acknowledge certain limitations. The study, relying on data from a single insurance company, might not be generalizable to populations covered by different insurance providers or to uninsured individuals. Moreover, although the analysis controlled for significant medical comorbidities, the study did not include medications used to treat these illnesses as covariates, potentially overlooking drug–drug interactions.

### 5.1. Future implications

The outcomes of our analysis suggest that metformin does not play a role in the prophylaxis or treatment of AMD. These findings may serve as a basis for encouraging additional prospective clinical trials to delve into the therapeutic potential of metformin in managing this particular patient group.

### 5.2. Strength and limitations

The strengths of this study are that the overall quality was good in most of the included studies in our analysis. Also, our meta-analysis included a variety of population-based studies including people with various ethnicities. Moreover, some papers have adjusted for confounding factors such as Lee et al.^[27]^ However, most of our studies were observational rather than randomized clinical trials. Therefore, prospective multicenter studies with larger sample sizes and longer follow-up durations are needed to support our findings and further evaluate the effect of metformin consumption on AMD.

## 6. Conclusion

Based on our findings, there is no statistically significant difference observed between the metformin group and non-metformin group concerning AMD. Similarly, no significant difference was identified between the AMD group and the control group regarding metformin usage. Therefore, we recommend that metformin should not be considered as a prophylactic measure or treatment for AMD. To strengthen and validate these conclusions, we emphasize the necessity for prospective randomized clinical trials.

## Author contributions

**Conceptualization:** Rowan H. Elhalag.

**Data curation:** Marwan Abowafia.

**Formal analysis:** Mai Saad Mohamed, Nada Mahmoud.

**Investigation:** Rowan H. Elhalag.

**Methodology:** Rowan H. Elhalag, Marina Ramzy Mourid.

**Project administration:** Rowan H. Elhalag, Jaffer Shah.

**Resources:** Rowan H. Elhalag, Momen Hassan Moussa.

**Software:** Marwan Abowafia.

**Supervision:** Karam R. Motawea.

**Validation:** Youmna Abourady.

**Visualization:** Paula Ghali.

**Writing – original draft:** Rowan H. Elhalag, Marwan Abowafia.

**Writing – review & editing:** Rowan H. Elhalag.

## Supplementary Material


