# Supplementary material for: The role of oral metformin in preventing and treating age-related macular degeneration: A meta-analysis
Source: Medicine (Baltimore). 2024 Jul 12;103(28):e38728. doi: 10.1097/MD.0000000000038728 (PMC11245258; doi:10.1097/MD.0000000000038728)
Supplement: Supplementary file 1 [file medi-103-e38728-s001.docx]

| **NOS scale Risk of Bias Assessment for case control studies** | | | | | | | | | | | |
| --- | --- | --- | --- | --- | --- | --- | --- | --- | --- | --- | --- |
|  |  | Selection | | | | Comparability | Exposure |  |  | Total Score | AHRQ standards |
| NO | Study | case definition | Representativeness | Selection of controls | Definition of controls | Comparability | Ascertainment | Same method | Non-response rate | Total Score | AHRQ standards |
| 1 | Blitzer,2021 | 1 | 1 | 1 | 1 | 1 | 1 | 1 | 1 | 8 | good |
| 2 | Brown, 2019 | 1 | 1 | 1 | 1 | 1 | 1 | 1 | 1 | 8 | good |
| 3 | Lee, 2019 | 1 | 1 | 1 | 1 | 1 | 1 | 1 | 1 | 8 | good |
| 4 | Shaw, 2022 |  |  | 1 |  | 1 | 1 | 1 |  | 4 | poor |
| 5 |  |  |  |  |  |  |  |  |  |  |  |
| 6 |  |  |  |  |  |  |  |  |  |  |  |
| 7 |  |  |  |  |  |  |  |  |  |  |  |
| 8 |  |  |  |  |  |  |  |  |  |  |  |
| 9 |  |  |  |  |  |  |  |  |  |  |  |
| 10 |  |  |  |  |  |  |  |  |  |  |  |
| 11 |  |  |  |  |  |  |  |  |  |  |  |
| 12 |  |  |  |  |  |  |  |  |  |  |  |
| 13 |  |  |  |  |  |  |  |  |  |  |  |
| 14 |  |  |  |  |  |  |  |  |  |  |  |
| 15 |  |  |  |  |  |  |  |  |  |  |  |
| 16 |  |  |  |  |  |  |  |  |  |  |  |
| 17 |  |  |  |  |  |  |  |  |  |  |  |
| 18 |  |  |  |  |  |  |  |  |  |  |  |
| 19 |  |  |  |  |  |  |  |  |  |  |  |

**



**

| **NOS scale Risk of Bias Assessment for cross-sectional studies** | | | | | | | | | | | |
| --- | --- | --- | --- | --- | --- | --- | --- | --- | --- | --- | --- |
|  |  | Selection | | | | Comparability | OUTCOME |  |  | Total Score | AHRQ standards |
| NO | Study | Representativeness | Non-respondents | Sample size | Ascertainment of t he exposure | Comparability | Assessment of outcome | Statistical test |  | Total Score | AHRQ standards |
| 5 | Stewart,2020 | 1 | 1 | 1 | 0 | 2 | 2 | 1 |  | 7 | good |
|  |  |  |  |  |  |  |  |  |  |  |  |

| **NOS scale Risk of Bias Assessment for cohort studies** |
| --- |

|  |  | Selection | | | | Comparability | OUTCOME |  |  | Total Score | AHRQ standards |
| --- | --- | --- | --- | --- | --- | --- | --- | --- | --- | --- | --- |
| NO | Study | Representativeness of exposed | Representativeness of non exposed | Ascertainment of exposure | outcome present or not | Comparability | Assessment of outcome | follow up long enough | adequacy of follow up | Total Score | AHRQ standards |
| 6 | Chen,2019 | 1 | 1 | 1 | 1 | 1 | 1 | 1 | 1 | 8 | good |
| 7 | Eton, 2022 | 1 | 1 | 1 | 1 | 1 | 1 | 1 | 1 | 8 | good |
| 8 | Gokhale,2022 | 1 | 1 | 1 | 1 | 1 | 1 | 1 | 1 | 8 | good |
| 9 | Jiang,2022 | 1 | 1 | 1 | 1 | 1 | 1 | 1 | 1 | 8 | good |
| 10 | vergroesen,2022 |  |  | 1 |  | 1 | 1 | 1 |  | 4 | poor |
